# Treatment of the behavioral variant of frontotemporal dementia: a narrative review

**DOI:** 10.1590/1980-57642021dn15-030004

**Published:** 2021

**Authors:** Leandro Boson Gambogi, Henrique Cerqueira Guimarães, Leonardo Cruz de Souza, Paulo Caramelli

**Affiliations:** 1 Behavioral and Cognitive Neurology Research Group, Departamento de Clínica Médica, Faculdade de Medicina, Universidade Federal de Minas Gerais – Belo Horizonte, MG, Brazil.; 2 Postgraduate Program in Neurosciences, Departamento de Clínica Médica, Faculdade de Medicina, Universidade Federal de Minas Gerais – Belo Horizonte, MG, Brazil.

**Keywords:** behavior control, drug therapy, frontotemporal dementia, review, controle comportamental, farmacoterapia, demência frontotemporal, revisão

## Abstract

Frontotemporal dementia (FTD) is a progressive neurodegenerative disorder accompanied by behavioral and personality changes and/or language deterioration. Its behavioral variant (bvFTD) is the main clinical presentation.

**Objective:**

This study aims to investigate the treatment alternatives for bvFTD available so far.

**Methods:**

We conducted a narrative review of bvFTD treatment options. We used PubMed and Lilacs databases with the terms “frontotemporal dementia” or “behavioral variant frontotemporal dementia” combined with “treatment,” “pharmacological treatment,” or “disease-modifying drugs.”

**Results:**

The articles retrieved and selected in the research pointed out that there is no specific treatment approved for bvFTD so far. The current proposals are limited to handle the cardinal behavioral symptoms of the disorder. Disease-modifying drugs are under development and may be promising, especially in the monogenic presentations of FTD.

**Conclusions:**

There are numerous approaches to treat the core symptoms of bvFTD, most of them based on low-quality research. To date, there are no drugs with a disease-specific therapeutic recommendation for bvFTD. Treatments are often investigated guided by primary psychiatric disorders with similar symptoms and should be chosen by the predominant symptom profile.

## RESUMO

A demência frontotemporal (DFT) é um transtorno neurodegenerativo progressivo acompanhado de deterioração do comportamento e da personalidade e/ou da linguagem. A variante comportamental (DFTvc) é a principal apresentação clínica.

**Objetivos:**

Investigar as alternativas de tratamento disponíveis para a DFTvc até o momento.

**Métodos:**

Realizou-se uma revisão narrativa das opções de tratamento da DFTvc. Os bancos de dados PubMed e Lilacs foram utilizados com os termos “demência frontotemporal” ou “variante comportamental da demência frontotemporal” combinados com “tratamento”, “tratamento farmacológico” ou “drogas modificadoras de doença”.

**Resultados:**

Os artigos recuperados e selecionados na pesquisa indicaram que não há nenhum tratamento específico aprovado até o momento para DFTvc. As propostas atuais são limitadas ao tratamento dos sintomas comportamentais cardinais do transtorno. As drogas modificadoras de doença estão em desenvolvimento e podem ser promissoras, especialmente nas apresentações monogênicas da DFT.

**Conclusões:**

Há inúmeras abordagens para tratar os principais sintomas DFTvc, a maioria delas baseada em pesquisas de baixa qualidade. Até o momento, não existem medicamentos com uma recomendação terapêutica específica para a DFTvc. Os tratamentos são frequentemente investigados guiados por distúrbios psiquiátricos primários com sintomas semelhantes e devem ser escolhidos pelo perfil de sintomas predominante.

## INTRODUCTION

Frontotemporal dementia (FTD) is a neurodegenerative disorder typically associated with progressive behavioral and personality changes and/or language deterioration. The first FTD diagnostic criteria were proposed by a consortium of researchers in Lund (Sweden) and Manchester (England) in 1994.^1^ These criteria were further refined by an international consensus in 1998.^2^ On that occasion, the term “frontotemporal lobar degeneration (FTLD)” was recommended for a broad spectrum of clinical conditions, from those with predominant behavioral changes to those with predominant language symptoms (primary progressive aphasia). Subsequently, the term FTLD was reserved for histopathological diagnosis. The clinical criteria for the diagnosis of behavioral variant (bvFTD) were revised in 2011, resulting in improvements in sensitivity.^3^

FTLD is considered as the second most frequent cause of early-onset dementia, with a prevalence estimated at 22 per 100,000 person-years among individuals aged between 45 and 64 years;^4,5^ about 25–30% of the cases occur in individuals aged 65 years or older.^6^ bvFTD constitutes the most common presentation form with the highest rate of inheritance and earlier onset, followed by language presentations. Around 40–50% of FTD cases present a positive family history, with 10% showing an autosomal dominant pattern.^7^ The most common monogenetic inheritance occur in microtubule-associated protein tau (*MAPT*), chromosome 9 open reading frame 72 (*C9ORF72*), and progranulin (*GRN*) genes, representing 25% of FTD cases.^8^

Since 2011,^3^ a bvFTD clinical presentation can be verified in the presence of progressive behavioral changes and/or cognitive deterioration, characterized by at least three of the six possible core symptoms: (1) social disinhibition; (2) apathy; (3) loss of sympathy or empathy; (4) persevering, stereotyped, or compulsive behavior; (5) hyperorality and dietary changes; and (6) neuropsychological profile characterized by executive dysfunction, with relative preservation of episodic memory and visuospatial abilities. The diagnosis is considered potential when there is a functional impairment as well as a frontal and/or temporal atrophy, evidenced by structural neuroimaging, and/or perfusion/metabolism deficits in the above-mentioned topography according to the functional neuroimaging studies. A definitive diagnosis of bvFTD is reserved only for cases with histopathological confirmation and/or the presence of a known pathogenetic mutation.^3^

Those core diagnostic symptoms of bvFTD result in significant stress to families and caregivers,^9^ and frequently these patients exhibit risky and even criminal behaviors.^10^ Therefore, the search for treatments that can attenuate symptoms or prevent progression of disease is of major relevance. To date, there is no effective disease-modifying therapy, and merely a few small studies have shown benefit of symptomatic pharmacological approaches. The design of interventional studies for FTD is challenging; there is low prevalence of FTD in the general population, lack of recognized diagnostic biomarkers, multiple clinical phenotypes, and genetic/pathological heterogeneity.

Many drugs, with distinct pharmacodynamic and pharmacokinetic characteristics, have been investigated as treatments for different symptoms of bvFTD. In addition, a variety of tools and scales with diverse psychometric properties have been used to measure the effects of these tested drugs. Trieu et al.^11^ have covered this topic in a recent systematic review. They included 23 studies reporting a total of 573 subjects and provided an overview of all the pharmacological treatment approaches that were taken targeting the main symptoms of bvFTD. It is a highly informative publication, but with a more complex text and harder consultation. In contrast, this article proposes leaner orientations that could guide clinical practice based on hypothetical theory on the mechanism of action of the drugs on each symptom. We hence offer a narrative review of the main approaches to treat this clinical condition.

## METHODS

We conducted a narrative review of the literature during 2020 through Lilacs and PubMed databases, using the words “treatment,” “pharmacological treatment,” and “disease-modifying drugs,” all of them combined with “frontotemporal dementia” or “behavioral variant frontotemporal dementia.” When indicated, other bibliographies were consulted from the reference lists of these articles. Only articles published in English or Portuguese were eligible, and there was no selection based on the publication date. The titles and abstracts of all the articles retrieved were read to identify the articles that addressed the topic and the purpose of this review.

## RESULTS

### Pharmacological treatment: overview

In sum, the articles retrieved pointed out that there are no approved drugs with specific indication for bvFTD. Pharmacological treatment is so far symptomatic, based on the case reports, small cases series, and a few open or controlled clinical trials (Table 1).

**Table 1.** Pharmacological treatment options for behavioral variant frontotemporal dementia.

Design	Authors	n	Drugs	Major findings
RCT	Moretti et al.^12^	16	Paroxetine 20 mg/day (n=8) vs. Piracetam 1,200 mg/day (n=8)	Significant improvements in different behavioral symptoms, reducing caregiver stress in the paroxetine group.
RCT	Deakin et al.^13^	10	Paroxetine 40 mg/day vs. placebo (crossover)	No improvement in cognition or behavior.
RCT	Lebert et al.^14^	26	Trazodone 150–300 mg/day vs. placebo (crossover)	Significant decrease in NPI scores, mediated by improvements in irritability, agitation, depression, and eating disorders in the trazodone group.
RCT	Rahman et al.^15^	8	Methylphenidate 40 mg/day vs. placebo (crossover)	Improvements in risk-taking behavior on a laboratory measurement of decision-making, but without effects on cognition.
RCT	Huey et al.^16^	8	Dextro-amphetamine 20 mg/day vs. quetiapine 150 mg/day (crossover)	Improvements in behavioral symptoms were noted for dextroamphetamine group, especially in disinhibition and apathy.
RCT	Kertesz et al.^17^	36	Galantamine 16–24 mg/day vs. placebo (crossover)	No significant improvements were noted in behavior or language for the whole group
RCT	Vercelletto et al.^18^	49	Memantine 20 mg/day (n=23) vs. placebo (n=26)	No improvements in the memantine group.
RCT	Jesso et al.^19^	20	Oxytocin 24 IU vs. placebo (crossover)	Improvement in apathy and expressions of empathy, resulting in better patient–caregiver interactions.
RCT	Boxer et al.^20^	81	Memantine 20 mg/day (n=39) vs. placebo (n=42)	No improvements in the memantine group.
RCT	Finger et al.^58^	23	Oxytocin 24, 48, or 72 IU (n=16) vs. placebo (n=7)	Trends of improvement were observed for the oxytocin-treated group, including apathy and empathy.
RCT	Pardini et al.^21^	8	Fortasyn Connect™ 125 mg/day vs. placebo (crossover)	Significant reduction in agitation, apathy, disinhibition, and irritability domains of NPI and an increase in the Theory of Mind skills compared to placebo.
RCT	Callegari et al.^22^	24	Agomelatine 50 mg/day vs. melatonin (crossover)	Significant apathy improvement in the agomelatine group.
Open-label studies	Swartz et al.^23^	11	Fluoxetine, sertraline, or paroxetine	Disinhibition, depressive symptoms, carbohydrate craving, and compulsions showed improvement in at least half of the subjects in which they had been present.
Open-label studies	Adler et al.^24^	6	Moclobemide	Minor improvement in the stereotypy of speech, echolalia, perseveration, elevated mood, inappropriate jocularity, aggressive behavior, irritability, and motor stereotypes.
Open-label studies	Ikeda et al.^25^	15	Fluvoxamine 50 mg/day	Improvement in stereotyped behavior and compulsive complaints of pain.
Open-label studies	Moretti et al.^26^	20	Rivastigmine 3–9 mg/day	Reductions in NPI, behavioral pathology in Alzheimer’s Disease Rating Scale, and Cornell Scale for Depression in Dementia scores, reduced caregiver burden, and stabilization in executive functions were seen in rivastigmine group.
Open-label studies	Mendez et al.^27^	8	Sertraline 50–100 mg/day	Decreased stereotypical movements.
Open-label studies	Diehl-Schmid et al.^28^	16	Memantine 20 mg/day	No changes in NPI or Frontal Behavioral Inventory were seen. ADAS-Cog scores increased, reflecting cognitive decline.
Open-label studies	Boxer et al.^29^	21	Memantine 20 mg/day	Transient improvement in NPI scores was seen predominantly in the bvFTD group.
Open-label studies	Hermann et al.^30^	15	Citalopram 30 mg/day	Improvement in NPI scores, especially irritability, disinhibition, and depression.
Case series	Moretti et al.^31^	3	Selegiline 1.25 mg/day	Improvement in NPI total score after 3 months.
Case series	Moretti et al.^32^	68	Olanzapine 2.5–10 mg/day	Reduction in delusions, social misconduct, wandering, and irritability.
Case series	Swanberg^33^	3	Memantine 20 mg/day	All subjects had improvement in total NPI score with specific improvements in apathy, agitation, and anxiety domains.
Case series	Mendez et al.^34^	12	Donepezil 10 mg/day	Worsening of symptoms according to the FTD Inventory. Four out of 12 treated subjects had increased disinhibition and compulsivity.
Case series	Czarnecki et al.^35^	3	Risperidone, olanzapine, and quetiapine	Subjects developed parkinsonism and tardive antecollis.
Case reports	Curtis et al.^36^	1	Risperidone	General improvement in psychotic symptoms and agitation.
Case reports	Goforth et al.^37^	1	Methylphenidate	Partial normalization of frontotemporal asymmetry (left > right) in QEEG slow-wave activity after methylphenidate administration. Patient had significant behavioral improvement.
Case reports	Fellgiebel et al.^38^	1	Aripiprazole 10 mg/day	Frontal lobe glucose metabolism declined over 12 months with conventional treatment, but improved after one month of aripiprazole.
Case reports	Cruz et al.^39^	1	Topiramate 200 mg/day	Reduced alcohol abuse, but not other obsessive behaviors.
Case reports	Poetter et al.^40^	1	Carbamazepine 800 mg/day	Sexual misconduct completely abated and never recurred over the subsequent 6 months.
Case reports	Singam et al.^41^	1	Topiramate 100 mg/day	Improvement in his eating behaviors; patient started to eat more slowly, stopped looking for sweets, and his wife no longer needed to hide food.

RCT: randomized controlled trial; ADAS-Cog, Alzheimer’s Disease Assessment Scale-Cognitive Subscale; bvFTD, behavioral variant frontotemporal dementia; FTD, frontotemporal dementia; NPI, neuropsychiatric inventory; QEEG, quantitative electroencephalography.

Unlike Alzheimer’s disease (AD), there is a relative preservation of the cholinergic system in bvFTD,^42^ and despite the lack of expectation, some cholinesterase inhibitors (ChEI) have been tested in RCT,^17^ open-label trial,^26^ and case series.^34^ Except in an open-label study with rivastigmine,^26^ these drugs did not lead to improvement in cognition or behavior17 and even worsened the symptoms in patients with bvFTD in a case series.^34^ Memantine has also been studied, and the results were equally negative, including two RCTs.^18,28,29,33^ Regardless, about 40% of patients diagnosed with bvFTD still receive ChEI or memantine at some point during their treatment according to the data collected in California, USA, and Girona, Spain.^43,44,45^

The most important abnormalities in bvFTD occur in dopaminergic and, especially, serotoninergic systems.^42^ Thus, the few studies with positive results grounded on better level of evidence, including three RCTs^12,13,14^ and two open-label studies,^23,25,27^ pertain to drugs acting on monoaminergic systems, especially serotonergic medications, such as the selective serotonin reuptake inhibitors (SSRI). Two small RCTs and one case series have also evaluated psychostimulant medications in bvFTD, pointing to subtle improvements in apathy, disinhibition, and abnormal risk-taking behavior, but the results were not replicated in studies with larger sample sizes.^15,16,37^

Moclobemide, a monoamine oxidase inhibitor (MAOI), has shown minor improvement in behavior, speech, and motor stereotype in an open-label study with six subjects.^24^ Selegiline, another MAOI, was found to improve the behavioral symptoms of three patients with probable FTD in a case series.^31^ The new antidepressant agomelatine improved apathy compared with melatonin in bvFTD patients.^21^

Whereas antipsychotics are commonly used to manage challenging behaviors, the rationale for their use in bvFTD emerges chiefly from uncontrolled case series and clinical reports.^35,38,44,46^

### Pharmacological treatments according to drug class

A practical way to assess the evidence regarding bvFTD symptomatic treatment is through drug class (Table 2).

**Table 2.** Pharmacological treatment of frontotemporal dementia symptoms through drug-class therapeutic response.

Drug class	Results	Studies’ design
Antidepressants (mostly SSRI and trazodone)	Improvement in behavioral symptoms	Case reports and series, open label trials, randomized double-blind placebo-controlled trial^12,13,14,23,25,27^
Atypical antipsychotics: risperidone, aripiprazole, olanzapine, quetiapine	Improvement in behavioral symptoms	Case reports and series^35,38,44,46^
Antiepileptics	Improvement in behavioral symptoms	Case reports and series^39,40,41^
Cholinesterase inhibitors	No improvement in cognition and worsening of behavioral symptoms	Open label trials, randomized, double-blind placebo controlled trials^17,26,34^
NMDA-antagonist (memantine)	Worsening of cognition and behavioral symptoms	Case series, open label trials, randomized double-blind placebo-controlled trials^18,28,29,33^

FTD: frontotemporal dementia; NMDA: *N*-methyl-d-aspartate.

Antidepressants, especially SSRIs, have been tested for the symptomatic treatment of bvFTD. Paroxetine, citalopram, fluvoxamine, and trazodone improved behavioral symptoms, but not cognition. Sertraline decreased stereotypical movements, and moclobemide produced slight improvement in behavioral symptoms.^12,13,14,23,25,27^

Although antipsychotics are traditionally used to control challenging behaviors, the evidence supporting their use in bvFTD comes essentially from uncontrolled case series and clinical reports. Besides the increased cardiovascular risk associated with these medications, almost all antipsychotics involve the risk of extrapyramidal side effects, to which patients with bvFTD are especially susceptible.^35^ Quetiapine has been shown to be effective in reducing agitation in case series, but showed no significant changes in NPI in a small double-blind trial.^16^ Risperidone and aripiprazole have been reported to improve agitation and improper behaviors in case series.^35,36^ Olanzapine was reported to improve NPI score in 17 patients with bvFTD in an open-label trial.^32^

Psychostimulants, such as methylphenidate and dextroamphetamine, are another class of drugs that should be used with extreme caution for FTD behaviors, as adverse results are not uncommon.^47^ These drugs modulate dopamine and have shown potentially positive preliminary results in behavioral symptoms of dementia, especially apathy.^48^

There are only few case reports suggesting improvement with anticonvulsants in the behavioral symptoms of bvFTD.^39,40,41^ Hence, there is a lack of more supportive evidence to suggest the routine use of antiepileptic drugs for bvFTD behavioral symptoms. This class of agents has substantial side effects that should be considered regarding their potential benefits.^49^

### Pharmacological treatments: symptom-focused approach

The use of pharmacological approach toward core or most disturbing bvFTD symptoms constitutes a standard clinical practice.^11^ Core bvFTD symptoms amenable to pharmacological interventions with available evidence of benefit are disinhibition, compulsive/perseverative behavior, hyperorality, apathy, and loss of sympathy/empathy (Table 3).

**Table 3.** Pharmacological treatments through symptom-focused approach.

bvFTD core symptom	Treatment options	Studies design
Behavioral disinhibition	SSRI and trazodone	Case reports and series, open label trials, randomized double-blind placebo-controlled trial^12,13,14,23,25,27^
	Atypical antipsychotics: risperidone, aripiprazole, olanzapine, quetiapine	Case reports and series^35,38,44,46^
Compulsive/perseverative behavior	SSRI and trazodone	Case reports and series, open label trials, randomized double-blind placebo-controlled trial trial^12,13,14,23,25,27^
Hyperorality	Oxytocin, topiramate, SSRI, and trazodone	Case reports and series, open label trials, randomized double-blind placebo-controlled trial trial^12,13,14,19,23,25,27,41^
Loss of empathy/sympathy	Oxytocin and Fortasyn Connect™	Randomized, double-blind, placebo-controlled studies^19,21^
Apathy	Psychostimulants	Randomized, double-blind, placebo-controlled studies and case report^15,16,37,47^

bvFTD: behavioral variant frontotemporal dementia; SSRI: selective serotonin reuptake inhibitors.

#### Disinhibition

Disinhibition is one of the most stressful symptoms of bvFTD, encompassing impulsivity, hypersexuality, risk behavior, legal infringements, embarrassment, and personal exposure. There is a probable link between disinhibition and serotonergic neurotransmission disruption in bvFTD patients, which showed a marked decrease in serotonin 5HT_1A_ and 5-HT_2A_ receptor-binding potential.^50,51^ This is the theoretical basis for antidepressant response in this form of dementia, especially SSRIs. As said, based on low-quality studies in bvFTD and evidence on symptom control of other psychiatric conditions, antipsychotics could be used to manage challenging and hazardous behavior, but with caution due to extrapyramidal and cardiovascular risks.^44^

#### Compulsive and perseverative behaviors

Compulsive and perseverative behaviors are manifested by collectionism, motor or vocal stereotypies, repetitive or ritualistic behaviors, and perseveration. The therapeutic target is the serotoninergic system and the reduced level of 5HT_1A_ and 5HT_2A_ receptors, especially those in the orbitofrontal cortex (OFC).^52^ Decreased functioning of 5-HT_2A_ receptors in the OFC could lead to abnormalities that potentially explain OFC-striatal hyperactivity found in OCD imaging studies.^53^ This is possibly the reason why SSRIs and trazodone have been considered efficacious treatment options in studies with fair quality of evidence.^12,13,14,23,25,27^

#### Hyperorality

Hyperorality is characterized by dietary changes such as gluttony, binge, or indiscriminate eating, and increased sweet preference, alcohol, and cigarette abuse. Moreover, patients disclose oral exploration and consumption of inedible items, particularly in more advanced stages of dementia. Hyperorality in bvFTD probably results from the involvement of the posterior hypothalamus and severe loss of brain serotonin neurotransmission, especially in 5HT_1A_ receptor.^54^ 5HT_1A_ receptors are associated, among other behaviors, with impulsivity and appetite.^30^ Thus, serotoninergic anti-depressants may help manage hyperorality in bvFTD patients and are well tolerated.^12,25^ Lebert et al. demonstrated trazodone efficacy in a sample of bvFTD patients according to NPI scores, including dietary changes.^14^

Jesso et al. found that oxytocin was associated with reduced NPI scores, as well as appetite and eating abnormalities.^19^ The antiepileptic drug topiramate seems to be an option to treat hyperorality; however, caution should be taken regarding its deleterious effects on cognition.^41,55^

#### Apathy

There is extensive evidence, especially from non-human animal studies,^56^ that dopaminergic deficits result in less-motivated behavior, which resembles the behavior of patients with apathy.^57^ Thus, psychostimulants such as methylphenidate and dextroamphetamine are usually prescribed to treat apathy in bvFTD.^47^ A Cochrane paper suggested that methylphenidate was the only drug with positive response in apathy, although this evidence was primarily obtained in AD patients’ samples.48 Finger et al. investigated the use of intranasal oxytocin and suggested that it may improve the levels of apathy.^58^

#### Loss of empathy/sympathy

Loss of empathy is manifested as diminished social interest and response to other people’s needs and feelings. Jesso et al. found that oxytocin was associated with reduced identification of negative facial expressions (anger and fear).^19^ It has been hypothesized that reduced perception of negative facial expressions may improve cooperative behavior. Finger et al. also found that intranasal oxytocin may promote expressions of empathy.^58^

Fortasyn Connect™, a nutraceutical compound thought to positively enhance synaptic function, was studied in 24 bvFTD subjects. Patients were enrolled and randomized to Fortasyn Connect™ (125 mL/day) or placebo groups. This nutraceutical was found to reduce behavioral deficits and improve social cognition, as demonstrated by an increase in the Theory of Mind skills compared with placebo.^21^ However, this was a proof-of-concept study, with a small number of subjects. It represents early-stage data that have not been confirmed by another research.

#### Parkinsonism

Many patients with bvFTD display parkinsonism, including trembling, stiffness, movement difficulties, and bradykinesia. Regrettably, parkinsonism in bvFTD is not well manageable. Patients are minimally responsive to levodopa. Treatment of parkinsonism, therefore, should be oriented by standard practices established for the management of idiopathic Parkinson’s disease (levodopa and dopaminergic agonists). Whether this can exacerbate behavioral symptoms in bvFTD is still unknown.^59^

### Non-pharmacological treatments

The current quality of evidence regarding non-pharmacological interventions for the behavioral symptoms of dementia is weak. In general, there is still a lack of strong and conclusive evidence on which non-pharmacological interventions should be specifically chosen by health professionals who care for elderly patients with behavioral symptoms over the care range. A recent review found that overall the music therapy had moderate effects on anxiety and minor effects on depression and behavioral symptoms in people with dementia. However, bvFTD cases were not included in this study.^60^

The tailored activity program (TAP) is an occupational therapy intervention for individuals with dementia and their caregivers, developed in eight home sessions over a period of 3–4 months. It has proven to be an acceptable intervention for individuals with bvFTD and their families. The qualitative analysis identified five themes: benefits perceived by caregivers, willingness of caregivers to change, strategies used by caregivers to engage people with dementia, barriers to implement TAP, and engagement of people with dementia. The quantitative analysis revealed a significant reduction in behavioral symptoms and maintenance of functional performance at the end of a 4-month trial.^61^

### Future perspectives and disease-modifying drugs

It is challenging to differentiate the various pathological conditions underpinning FTD, and the absence of reliable biomarkers has been a challenge in the search for disease-modifying drugs.^62,63^ Regardless, there is an increased understanding in the field, and there are ongoing clinical trials with drugs with potential disease-modifying effect. Currently, the main therapeutic targets are preventing tau aggregates (aggregation inhibitor, acetylation inhibitor, and immunotherapies for aggregates reduction); preventing tau loss of normal function; clearing the transactive DNA-binding protein 43 (TDP43) aggregates; preventing Fused in sarcoma, Ewing sarcoma, and TATA-box-binding associated factor 15 (FET)-protein family accumulation; raising or restoring progranulin levels; and suppressing the expression of harmful genes.^62,63^ Several of those potential disease-modifying therapies have been or are being tested in Phase II clinical trials. So far, there were negative outcomes of Phase III study of *leuco-methyl thioninium*, trx0237 (LMTM), a drug for the aggregation of tau protein.^62,63^

There are several approaches to treat bvFTD core symptoms, most of them based on low-quality research. There is no medication with specific therapeutic indication for bvFTD so far.

Treatments and neurotransmission disturbances in bvFTD are often investigated based on primary psychiatric disorders with similar symptoms, such as the compulsive behavior of obsessive–compulsive disorder or the agitation and psychotic symptoms of schizophrenic and bipolar disorders, and pharmacological treatment should be guided by the predominant symptoms profile. Thus, SSRIs and trazodone are medications that can be useful in the control of some behavioral symptoms such as disinhibition, persevering, stereotyped behaviors, and hyperorality. Nevertheless, apathy, a common symptom in bvFTD, does not usually respond to these medications, and psychostimulants could be an option in some cases. Antipsychotics should be avoided as the evidence is weak and the risks are high. There are non-pharmacological interventions, especially music therapy and training of family members and caregivers, which should be offered whenever possible. Clinical trials with drugs that have potential disease-modifying effects on FTD are still in progress. Moreover, promising therapies directed to monogenic forms of FTD are also under investigation.
